# Internet of Things–Enabled Technologies for Weight Management in Children and Adolescents: Protocol for a Systematic Review

**DOI:** 10.2196/16930

**Published:** 2020-03-31

**Authors:** Ching Lam, Madison Milne-Ives, Michelle Helena Van Velthoven, Edward Meinert

**Affiliations:** 1 Digitally Enabled Population Health Research Group Department of Paediatrics University of Oxford Oxford United Kingdom; 2 Department of Primary Care and Public Health Imperial College London United Kingdom

**Keywords:** Internet of Things, IoT, childhood obesity, wearables, physical activity tracking

## Abstract

**Background:**

Childhood obesity is a serious global issue, leading to increased medical spending on obesity-related diseases such as cardiovascular diseases and diabetes. There is a need for health care services that link health behavior to risk factors, such as diet and physical activity, and that provide better advice and feedback to users, which Internet of Things–enabled technologies could facilitate.

**Objective:**

The objective of the systematic review will be to identify available Internet of Things–enabled technologies for weight management of children and adolescents (users younger than 18 years). It will also aim to understand the use, effectiveness, and feasibility of these technologies.

**Methods:**

We will search the Medline, PubMed, Web of Science, Scopus, ProQuest Central, and IEEE Xplore Digital Library databases for studies published after 2010, using a combination of keywords and subject headings related to health activity tracking, youth, and Internet of Things. In addition, a Google search to identify grey literature will be conducted. Two authors will independently screen the titles and abstracts identified from the search and accept or reject the studies according to the study inclusion criteria. Any discrepancies will then be discussed and resolved. The quality of the included studies will be assessed using the Critical Appraisal Skills Programme (CASP) checklists. Data from included studies will be extracted into a predesigned form to identify the types of devices or apps, Internet of Things applications, and health outcomes related to weight management.

**Results:**

A preliminary search on Medline returned 484 results. The publication of the final systematic review is expected in mid-2020.

**Conclusions:**

The effectiveness and feasibility of physical activity trackers and consumer wearables for different patient groups have been well reviewed, but there are currently no published reviews that look into these technologies in the wider Internet of Things context. This review aims to address this gap by examining Internet of Things–enabled technologies that are designed for youth weight management and thus inform further research and clinical studies to reduce childhood obesity.

**International Registered Report Identifier (IRRID):**

PRR1-10.2196/16930

## Introduction

### Background

Childhood obesity and related costs are increasing worldwide [1-3]. It has been shown that the majority of children with obesity remained obese in adulthood, resulting in significant health care costs [4]. Promoting better diet and regular physical activities [5] can help prevent childhood obesity and thus reduce the occurrence of obesity-related metabolic and cardiovascular diseases [6], and in turn reduce health care costs.

Management of childhood obesity usually involves patient lifestyle counseling [7], but face-to-face counseling can be expensive and difficult for patients in rural areas [5]. There is a growing emphasis on patient-centered health care, where care extends beyond the hospital and is “respectful of and responsive to individual patient preferences, needs, values, and ensuring that patient values guide all clinical decisions”[8]. A promising solution for patient-centered care is the deployment of connected health technologies for remote diagnosis, monitoring, and treatment and patient self-care and support [9,10]. The tracking of physical activity has been studied extensively since the 2000s [11]. It is only in the previous 5 years that wearables, such as wristwatches with activity sensors, have become sophisticated enough to provide measures beyond steps, distance, calories, and sleep. For example, wearables can now measure activity minutes, heart rate, and goal and target-oriented designs [12]. These advances in wearables allow more accurate tracking of physical activities and provide greater insight into the type and form of physical activity undertaken by the user, such as exercise intensity and metabolic rate [13].

The use of these wearables can further be enhanced through integration with the Internet of Things (IoT) [14]. Since the advent of IoT in 1999 [15], IoT-enabled devices and networks have been applied to a range of applications including smart cities [16], virtual power plants [17], and health care [18,19]. In the health care setting, IoT-enabled devices—technologies that are connected to a network–can give accurate and real-time feedback that can generate health data to improve understanding of user behavior and personalize treatment regimes. Acampora et al [20] surveyed the applications of IoT in health care and identified six main applications in health care (Table 1).

**Table 1:** Summary applications of Internet of Things–enabled technologies in health care.

Application	Goal
Health monitoring, behavioral monitoring, emergency detection	Sensor networks for physiological measures (electrocardiogram, electroencephalogram, etc), health behaviors (such as physical activity) and hazard detection (such as falls)
Assisted living	Smart environments are created to support patients and older adults in their daily lives
Therapy and rehabilitation	Remote and autonomous rehabilitation service provision
Persuasive well-being	Motivate users to adopt a healthier lifestyle
Emotional well-being	Analyze emotions and improve mental well-being of users
Smart hospitals	Improve communication among hospitals

Technologies that track physical activity are widely used in health care applications. Previous studies have reviewed different applications of IoT and non-IoT physical activity trackers for various patient groups, including patients with rheumatic and musculoskeletal diseases [21], epilepsy, Parkinson, patients who have had a stroke [22], working-age women [23], people with serious mental illness [24], obese adults [25], and children [26,27]. These wearable activity trackers have generally shown significant effectiveness in increasing physical activity in the short-term [28], but there are still relatively few studies justifying their long term effectiveness [29]. Furthermore, long-term user adherence remains challenging [30].

Most systematic reviews on physical activity tracking and weight management in children were published several years ago. However, one recent review examined the feasibility and effectiveness of wearable activity trackers for young people (5-19 years old) [27]. Overall, they found that measures of effectiveness were mostly positive but nonsignificant. However, this review included any type of wearable activity tracker and did not focus on IoT.

With regard to user perspectives of mobile health interventions for weight management, it was found that user acceptance of trackers can vary greatly depending on their age group [31,32]. In addition, although the data from wearables can be a valuable source of data for diagnosis and patient monitoring, they are oftentimes not standardized or validated, which limits their clinical significance in supporting clinical decision making [22,33].

### Objectives

There is a gap in the literature regarding children and adolescent’s use of digital weight management technologies that are connected to a wider network. Currently, no studies focusing on the wider context of IoT-enabled technologies for weight management in children and adolescents have been published. Previously published reviews have focused on elements of this (ie, weight management in children and adolescents, the use of IoT in weight management technologies), but none have examined the intersection. This review will aim to understand how IoT-enabled technologies designed for youth, such as physical activity trackers and weight management apps, fit in the wider IoT context, how data obtained through these devices can improve health care delivery, and whether these address privacy concerns by considering regulations. Specifically, the review aims to answer the following questions:

1. What IoT-enabled solutions are used for weight management and physical activity encouragement in children and adolescents (<18 years old)?
2. How are the data collected with IoT-enabled solutions analyzed, and how do the technologies connect and contribute to the wider IoT ecosystem?
3. What are the ethical and regulatory barriers in implementing these technologies, especially with regard to data sharing and privacy?
4. What are the effectiveness measures used and reported by researchers?

## Methods

### Protocol

This protocol is developed in accordance with the Preferred Reporting Items for Systematic Reviews and Meta-Analyses Protocols (PRISMA-P) statement [34] and the systematic review to be executed based on this protocol will also be conducted in accordance with PRISMA.

### Search

We will search Medline, Pubmed, Web of Science, Scopus, ProQuest Central, Engineering Village Compendex, and the IEEE Xplore Digital Library for studies published after 2010 using a combination of keywords and subject headings related to health activity tracking, youth, and IoT (Table 2). The search string will be constructed in this format: (Health activity tracking) AND (Youth) AND (IoT). Additionally, a Google search will be used to identify gray literature. A sample search conducted in Medline can be found in Multimedia Appendix 1.

**Table 2:** Search terms.

Theme	MeSH^a^ terms^b^	Search terms
Health activity–tracking device	Fitness Trackers	electronic track* OR (electronic activ* AND track*) OR (electronic activ* AND monitor*) OR electronic fitness track* OR fitness track* OR (wearable AND track*) OR wearable OR sens*
Weight management	Weight Reduction, Body Mass Index	(Weight AND (manag* OR monitor* OR reduc* OR loss OR maint*)) OR (“body mass index” OR BMI) OR diet OR obes*
Youth	Pediatrics, Child, Adolescent	Child* OR teen* OR youth OR paed* OR ped* OR adolescent* OR young*
Internet of Things	N/A^c^	IoT OR Internet of things OR connected health OR digital health OR mobile health OR mhealth OR Bluetooth OR wireless OR application processing interface OR API

^a^MeSH: Medical Subject Headings.

^b^MeSH terms were included in the search terms and are only identified separately in this table to describe which search terms were MeSH terms.

^c^N/A: not applicable.

### Eligibility Criteria

The study was defined as follows using the PICO (population, intervention, comparator, outcomes) model:

- Population: Children and adolescents younger than 18 who have interacted with IoT-enabled technologies for weight management, physical activity tracking, and encouragement of a healthy lifestyle.
- Intervention: Wearable IoT-enabled weight management and tracking technologies (eg, physical activity tracker, food tracker, sleep tracker) and other physical activity or dietary interventions designed to help young people lose or maintain weight.
- Comparator: Studies with and without a comparator will be included.
- Outcomes: This study will provide information on (1) the available weight management technologies and products for young people (designed for users below the age of 18), (2) the reported effectiveness of these technologies and the measures used to assess them, and (3) the benefits and limitations of each of these technologies reported by the studies.

The inclusion and exclusion criteria for the study can be found in Textbox 1.

### Study Records

#### Data Management and Selection Process

All search results will be exported into a Mendeley library and duplicates will be removed. Two authors (CL and MM-I) will independently screen the titles and abstracts identified from the search and accept or reject the studies according to the study inclusion and exclusion criteria. Any discrepancies will then be discussed and resolved. The full texts will be downloaded for the selected studies and analyzed to determine eligibility. Where there is disagreement in either the screening or full-text analysis stages, a third reviewer will be consulted until consensus is reached.

#### Data Extraction

One reviewer will extract data from the included studies, which will be validated by a second reviewer. Data from eligible publications will be extracted into a predesigned form to identify the types of devices or apps, IoT architecture employed, effectiveness for youth weight management, and relevant ethics and regulations if mentioned (Textbox 2). The form was custom-built to reflect reported items identified in a preliminary search of the literature that were relevant to the stated research questions.

**Study inclusion and exclusion criteria.**

**Inclusion criteria**

- English publications
- Studies published between 2010 (year of first published study identified by Ridgers et al for wearable activity trackers for youth [27]) and present
- Studies that describe a wearable device or mobile app for health activity tracking connected to a wider network (including internet and other networks beyond the standalone device)
- Health activity tracking and other weight management devices for young people that have data analysis functionalities connected to a network
- Studies that describes the data analysis process and how data is connected to the wider network

**Exclusion criteria**

- Studies that do not describe weight management intervention
- Studies that describe devices or mobile apps that are not connected to a wider network/platform for data analysis
- Health activity tracking devices that are not connected to a network beyond simple data storage
- Studies that are not focused on children or adolescents (below 18 years of age)

**Data extraction by theme.**

**Background information of study**

- Year
- Country
- Target patient age group
- Test sample size
- Trial type
- Length of study
- Scientific theory

**Sensing layer**

- Product
- Type of device (eg, mobile app, wearable tracker)
- Sensor type
- Data collected (eg, activity, food intake, heart rate, sleep)

**Networking layer**

- Data transfer method

**Service/interface layer**

- What user needs does the product aim to satisfy
- Stakeholders (if identified)
- Data analysis methods

**Effectiveness**

- Researcher-reported effectiveness measures and outcomes (if any)

**Ethics and governance**

- Mention of ethics
- Mention of regulation

#### Data Analysis

As the number of studies are expected to be limited, the analysis will be qualitative, focusing on how existing technologies interact with IoT and connect to the wider internet.

#### Quality Appraisal

Two reviewers will independently review the identified randomized controlled trials using the risk of bias tool developed by the Cochrane Collaboration [35]. The quality of other included studies and systematic reviews will be assessed using relevant Critical Appraisal Skills Programme (CASP) checklists (eg, CASP checklist for randomized controlled trials, CASP checklist for systematic review) [36]. As we do not expect a large number of studies to meet the inclusion criteria, risk of bias and quality will be reported, but no studies will be removed.

## Results

This study aims to identify the types of IoT-enabled technologies used for weight management of children and adolescents and identify the benefits, effectiveness, and limitations of each of these technologies. A sample search conducted on PubMed returned 592 results (search string and results can be found in Appendix 1). The results will inform future development of weight management technologies for children and adolescents to reduce the cost burden of childhood obesity on health care systems. Full results will be published mid-2020.

## Discussion

Although wearables and apps for health activity management have been well-reviewed, their integration with the wider IoT network represents a gap in the knowledge for improving patient-centered health care. This systematic review will allow the health care and app development community to better understand how current technologies interact with the internet and provide insights for future IoT–enabled technology development for children and adolescents. The results of this work will inform future development of weight management technologies for young people. A potential limitation of this review is that studies of IoT devices might overrepresent children and adolescents from high socio-economic status, if the devices were preowned and not provided for the study.

This systematic review protocol will be executed within the next 12 months and the review will be published in a peer-reviewed journal to inform future developments in IoT-enabled childhood obesity management technologies.

## Appendix

Multimedia Appendix 1 Initial search results.

## Abbreviations

CASP: Critical Appraisal Skills Programme
IoT: Internet of Things
MeSH: Medical Subject Headings
PICO: population, intervention, comparator, outcomes
PRISMA-P: Preferred Reporting Items for Systematic Reviews and Meta-Analyses Protocols
