# Supplementary material for: Internet of Things–Enabled Technologies for Weight Management in Children and Adolescents: Protocol for a Systematic Review
Source: JMIR Res Protoc. 2020 Mar 31;9(3):e16930. doi: 10.2196/16930 (PMC7157501; doi:10.2196/16930)
Supplement: Multimedia Appendix 1 [file resprot_v9i3e16930_app1.docx]

Appendix

Medline search results

| Search string | Database | Results |
| --- | --- | --- |
| ((electronic track* or (electronic activ* and track*) or (electronic activ* and monitor*) or electronic fitness track* or fitness track* or (wearable and track*) or wearable or sens*) and (Weight or body mass index or BMI or diet or obes*) and (Child* or teen* or youth or paed* or ped* or adolescent* or young*) and (IoT or Internet of things or data analy* or data collect* or connected health or digital health or mobile health or mhealth)).af.  limit 1 to (yr="2010 -Current" and "all child (0 to 18 years)") | Medline | 484 |
